# A multi-biomarker disease activity score can predict sustained remission in rheumatoid arthritis

**DOI:** 10.1186/s13075-020-02240-w

**Published:** 2020-06-24

**Authors:** M. H. Y. Ma, N. Defranoux, W. Li, E. H. Sasso, F. Ibrahim, D. L. Scott, A. P. Cope

**Affiliations:** 1 Academic Department of Rheumatology, Division of Immunology, Infection and Inflammatory Disease, Faculty of Life Sciences and Medicine, King’s College London, Weston Education Centre, 10 Cutcombe Road., London, SE5 9RJ UK; 2 Level 10, Tower Block, Division of Rheumatology, University Medicine Cluster, National University Health System, 1E Kent Ridge Road, Singapore, 119228 Singapore; 3 Department of Medicine, Yong Loo Lin School of Medicine, National University of Singapore, Singapore, 119228 Singapore; 4 Crescendo Bioscience, Inc., South San Francisco, CA USA; 5 Present Address: Parker Institute for Cancer Immunotherapy, San Francisco, CA USA; 6 MyoKardia, Inc., Brisbane, CA USA; 7 Department of Rheumatology, Guy’s and St Thomas’ UK National Health Service (NHS) Foundation Trust, London, UK

**Keywords:** Rheumatoid arthritis, Remission, MBDA score, IL6, SAA, Leptin, CRP

## Abstract

**Background:**

Reliable assessment of remission is important for the optimal management of rheumatoid arthritis (RA) patients. In this study, we used the multi-biomarker disease activity (MBDA) test to explore the role of biomarkers in predicting point remission and sustained remission.

**Methods:**

RA patients on > 6 months stable therapy in stable low disease activity (DAS28-ESR ≤ 3.2) were assessed every 3 months for 1 year. Baseline, intermittent (IR) and sustained (SR) remission were defined by DAS28-ESR, DAS28-CRP, simple disease activity index (SDAI), clinical disease activity index (CDAI) and ACR/EULAR Boolean criteria. Patients not fulfilling any remission criteria at baseline were classified as ‘low disease activity state’ (LDAS). Patients not fulfilling any remission criteria over 1 year were classified as ‘persistent disease activity’ (PDA). MBDA score was measured at baseline/3/6 months. The baseline MBDA score, the 6-month time-integrated MBDA score and MBDA biomarkers were used for analyses. The area under the receiver operating characteristic curve (AUROC) assessed the ability of the MBDA score to discriminate between remission and non-remission. Biomarkers were analysed at baseline using the Mann-Whitney test and over time using the Jonckheere-Terpstra trend test.

**Results:**

Of 148 patients, 27% were in the LDAS, 65% DAS28-ESR remission, 51% DAS28-CRP remission, 40% SDAI remission, 43% CDAI remission and 25% ACR/EULAR Boolean remission at baseline. Over 1 year, 9% of patients were classified as PDA. IR and SR were achieved in 42%/47% by DAS28-ESR, 46%/29% by DAS28-CRP, 45%/20% by SDAI, 44%/21% by CDAI and 35%/9% by ACR/EULAR Boolean criteria, respectively. By all remission criteria, baseline MBDA score discriminated baseline remission (AUROCs 0.68–0.75) and IR/SR (AUROCs 0.65–0.74). The 6-month time-integrated MBDA score discriminated IR/SR (AUROCs 0.65–0.79). Baseline MBDA score and concentrations of IL-6, leptin, SAA and CRP were significantly lower in all baseline remission criteria groups vs LDAS. They and the 6-month time-integrated values were lower among patients who achieved IR/SR vs PDA over 1 year.

**Conclusions:**

This study demonstrated that the MBDA score and its biomarkers IL-6, leptin, SAA and CRP differentiated between small differences in disease activity (i.e. between low disease activity and remission states). They were also predictors of remission over 1 year.

## Introduction

Rheumatoid arthritis (RA) is a chronic disease with heterogeneous outcomes. Early treatment intervention, biologic therapies, and tight control treatment strategies have made achievement of low disease-activity, including remission states, increasingly common [1]. The remission criteria that have evolved over the last two decades reflect a common underlying theme. These criteria include measurement of clinical variables assessed by clinicians and patients, such as joint counts and global scores, as well as inflammatory blood markers, such as the erythrocyte sedimentation rate (ESR) or C-reactive protein (CRP). Despite attempts to define remission in many different ways [2–5], the definition of a true state of biological remission remains elusive. A substantial proportion of patients classified as being in clinical remission continue to develop radiographic progression [6–9]. In addition, imaging studies have shown that the majority of patients in remission, whether treated with conventional DMARDs or a biological agent, have evidence of active subclinical inflammation [10, 11]. Flares can occur when DMARDs are tapered or stopped for patients in low disease activity or clinical remission [12]. These findings indicate that a subset of patients in clinical remission have clinically significant disease despite displaying few clinical signs or symptoms.

The clinical components of the conventional disease activity assessments are subject to intra-assessor and inter-assessor variability [13, 14] and can be confounded by co-morbidities such as fibromyalgia and joint damage [15]. The biomarkers ESR or CRP have limitations because they are non-specific and can be elevated in a number of conditions, such as older age, anaemia, infection and malignancy, and they may also be normal in many patients with clinically active RA [16, 17]. These considerations highlight the need for identifying novel measures to complement clinical assessment of remission status.

A multi-biomarker disease activity (MBDA) score measures 12 serum protein biomarkers and has been validated as an objective measure of disease activity across a range of disease activity states [18, 19]. These 12 biomarkers reflect biological pathways involved in the pathogenesis of RA and can be broadly grouped as acute-phase reactants (SAA, CRP), hormones (leptin and resistin), growth factors (VEGF and EGF), adhesion molecules (VCAM1), skeletal-related proteins (YKL-40), matrix metalloproteinases (MMP-1, MMP-3) and cytokine-related proteins (IL-6, TNFR1). The MBDA score has been shown to predict risk for radiographic progression [20]. Therefore, these objective markers may be useful for defining remission on a molecular level and may have potential for predicting sustained remission. In post hoc analysis of OPERA, a study of patients with active recent-onset RA who were treated with methotrexate alone or with adalimumab, changes in MBDA score from baseline to 3 months were associated with achieving clinical remission at 6 months [21]. In contrast, the utility of the MBDA score for predicting outcomes in patients who are in stable low disease activity states while continuing a stable treatment has not been examined in depth.

Reliable assessment of remission is important for the optimal management of patients with RA to achieve the best clinical, radiographic and functional outcomes possible. We have addressed this need in the present study by evaluating a cohort of clinically similar patients in low disease activity or remission. We aimed to identify serum molecular markers of remission and to explore the role of these biomarkers in predicting point and sustained remission as defined by several clinical remission criteria.

## Methods and patients

### The REMIRA cohort

Adult RA patients diagnosed according to the 1987 revised ACR criteria were recruited into the REMIRA (REMission In RA) study [22, 23]. Three centres in South London participated in this study: Guy’s and St Thomas’ Hospital, King’s College Hospital and University Hospital Lewisham NHS Foundation Trusts. Inclusion criteria were (1) disease duration < 10 years, (2) stable treatment for ≥ 6 months and (3) stable low disease activity defined as DAS28-ESR ≤ 3.2 at two assessments at least 1 month apart.

### Ethics approval

The study was approved by the local ethics committee and conducted according to the guidelines of the Declaration of Helsinki. Written informed consent was obtained from all participants (REC:09/H0803/154, Wandsworth Research Ethics Committee).

### Clinical assessments

Clinical assessments were performed at baseline and months 3, 6, 9 and 12. Internationally accepted criteria were used [2–5]. Several definitions of remission were examined: DAS28-ESR < 2.6, DAS28-CRP < 2.3, SDAI ≤ 3.3, CDAI ≤ 2.8 and the ACR/EULAR Boolean remission, defined as TJC, SJC, CRP (mg/dl) and patient VAS (0–10 cm) all ≤ 1. For each definition, point remission at baseline was defined as meeting any of these definitions of remission and low disease activity state (LDAS) was defined as not fulfilling any of the definitions of clinical remission used in this study at baseline. Non-remission was defined as not being in remission by a given remission criterion at a given visit. Longitudinal analyses were also carried out. Sustained remission (SR) was defined as achieving remission by the same criterion at all visits from baseline to 12 months. Intermittent remission (IR) was defined as achieving remission by a criterion on at least 1 study visit from baseline to 12 months, but not all visits. Sustained non-remission was defined as not fulfilling a given remission criterion at any visit from baseline to 12 months. Persistent disease activity (PDA) was defined as never achieving remission by any clinical definition at any visit from baseline to 12 months.

### Serum biomarkers

Sera were collected for biomarker measurement at baseline, 3 months and 6 months using standard serum separator tubes according to the manufacturer’s instructions. Sera were frozen at − 80 °C within 6 h of blood sampling. Frozen serum samples were shipped to Crescendo Bioscience, South San Francisco CA, USA, for MBDA testing in their laboratory facility using the same instrument, reagents and algorithm used for the Vectra® test. The serum concentrations of 12 biomarker proteins (IL-6, SAA, CRP, EGF, VEGF, resistin, leptin, MMP-1, MMP-3, VCAM1, YKL-40, TNFR1) were measured and combined using a previously established, validated algorithm to provide a score that has a scale of 1–100 and pre-defined disease activity categories of low (< 30), moderate (30–44) and high (> 44) [18, 19]. MBDA scores in this study were determined with the validated MBDA algorithm [18, 19], without adjustment for age, sex or adiposity.

### Statistical analysis

STATA/SE 11.2 (StataCorp, College Station, TX, USA) was used for statistical analysis. Patient characteristics and baseline biomarker-related measurements were summarised by median values and interquartile ranges (IQR) for continuous variables, and by percentage of subjects in each category for categorical variables. To assess the relationship between biomarkers or MBDA score and remission states over 1 year, baseline values and time-integrated values were used. Time-integrated (ti) value was defined as the mean of the values at baseline, 3 months and 6 months. Clinical data were analysed as observed, without imputation for missing data. Missing biomarker data were imputed with last-observation-carried-forward (LOCF). The area under the receiver operating characteristic curve (AUROC) was used to assess the following: (1) the ability of baseline MBDA score to discriminate between baseline remission and baseline non-remission, (2) the ability of baseline MBDA score to discriminate between intermittent or sustained remission over 1 year (IR/SR) vs. sustained non-remission over 1 year and (3) the ability of time-integrated MBDA (tiMBDA) score to discriminate between IR/SR vs. sustained non-remission over 1 year, using five clinical composite definitions of remission (DAS28-ESR, DAS28-CRP, SDAI, CDAI and ACR/EULAR Boolean).

The difference in MBDA scores and biomarker concentrations between patients in remission compared to LDAS at baseline was assessed by the Mann-Whitney test. The distribution of MBDA scores or biomarker concentrations among PDA/IR/SR groups was assessed using the Jonckheere-Terpstra trend test, while the Mann-Whitney test was used to perform pairwise comparisons of MBDA scores between groups.

## Results

### Patient cohort

**Fig. 1:**
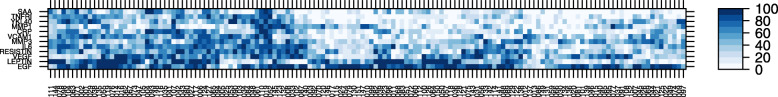
Heatmap demonstrating heterogeneity of the biomarker concentrations among patients at baseline. Patients are ordered across the *X*-axis based on hierarchical cluster analysis of the percentiles of the biomarker levels, for which the associated intensities are indicated in the greyscale legend at right. The Euclidean distance and Ward’s criterion were used for clustering

Serum samples were available for 148 patients from the REMIRA cohort. Table 1 shows the patient characteristics. The median age (IQR) was 58 years (48–69), median disease duration 40 months (23–77) and 66% were female. Eighty-three per cent were IgM RF-positive and 55% were ACPA-positive. At baseline, 87% of patients were being treated with methotrexate, 26% with sulfasalazine, 30% with hydroxychloroquine, 3% with leflunomide and 2% with prednisolone. Eighteen per cent were receiving anti-TNF therapy in combination with csDMARDs. As expected, the median DAS28-ESR, DAS28-CRP, SDAI and CDAI at baseline (2.08, 2.50, 4.1 and 3.7, respectively) were all in the low category. The median (IQR) baseline MBDA score was 31 (18–39) and the median tiMBDA score was 29 (20–39). The heatmap in Fig. 1 highlights the degree of heterogeneity of biomarker levels at baseline for all patients, who all had DAS28-ESR < 3.2 and, thus, had relatively similar levels of disease activity clinically.

**Table 1:** Baseline patient characteristics and biomarker levels (*N* = 148)

**Patient Characteristics**		
Age, median (IQR) years		58 (48, 69)
Disease Duration, median (IQR) months		40 (23, 77)
Female (percentage)		66%
IgM RF Positive (percentage)		83%
ACPA Positive (percentage)^1^		55%
Ethnicity	Caucasian	77%
	Asian	5%
	Afro-Caribbean	18%
TJC28, median (IQR)		0 (0, 1)
SJC28, median (IQR)		0 (0, 2)
ESR, median (IQR)		7 (4, 13)
Patient Global, median (IQR) (0–100 mm)		19 (9, 36)
DAS28-ESR, median (IQR)		2.08 (1.44, 2.77)
DAS28-CRP, median (IQR)		2.50 (2.13, 3.09)
SDAI, median (IQR)		4.1 (2, 8.23)
CDAI, median (IQR)		3.7 (1.5, 7.8)
Treatments	Methotrexate	87%
	Sulfasalazine	26%
	Hydroxychloroquine	30%
	Leflunomide	3%
	Prednisolone	2%
	Combination csDMARDs	51%
	Tumour necrosis factor inhibitors^2^	18%
**Biomarker**	**Baseline values (median, IQR)**	**Time-integrated values over 6 months (median, IQR)**
MBDA score (1–100)	31 (18–39)	29 (20–39)
EGF (pg/ml)	251.65 (171.13–360.64)	275.08 (173.97–358.55)
IL-6 (pg/ml)	9.73 (5.98–16.65)	10.37 (6.98–15.72)
Leptin (ng/ml)	11.78 (0.44–27.49)	11.04 (4.95–27.30)
MMP-1 (ng/ml)	6.80 (4.56–9.56)	7.09 (4.69–9.44)
MMP-3 (ng/ml)	25.38 (17.37–37.10)	25.23 (17.90–35.35)
Resistin (ng/ml)	7.42 (6.00–9.49)	7.61 (6.48–9.65)
SAA (μg/ml)	1.68 (0.95–3.17)	1.92 (0.87–3.97)
CRP (mg/l)	2.36 (1.10–6.14)	1.18 (2.43–5.71)
TNFR1 (ng/ml)	1.55 (1.34–1.99)	1.57 (1.38–2.00)
VCAM1 (ng/ml)	534.79 (437.82–639.31)	519.48 (431.13–610.21)
VEGF (pg/ml)	271 (177–389)	273 (189–404)
YKL-40 (ng/ml)	57.46 (36.70–92.16)	61.01 (36.82–101.60)

^1^ACPA data available for 94 patients

^2^All patients receiving a TNFi were receiving concomitant treatment with a csDMARD

### Baseline MBDA score and time-integrated MBDA score for discrimination of clinical remission by AUROC

The ability of the MBDA score at baseline to differentiate between remission and non-remission at baseline for the five clinical remission criteria is shown in Table 2. AUROC values ranged from 0.68 for CDAI remission to 0.75 for DAS28-ESR remission, with 95% CIs indicating statistical significance for each clinical remission criterion tested. Baseline MBDA score was also able to differentiate between intermittent or sustained remission (IR/SR) and sustained non-remission over 1 year for each remission criterion, with AUROC values ranging from 0.65 for ACR/EULAR Boolean remission to 0.74 for DAS28-CRP remission. The tiMBDA score (i.e. time averaged over 6 months) also significantly differentiated between IR/SR and sustained non-remission over 1 year for each of the remission criteria tested, with AUROC values ranging from 0.65 for ACR/EULAR Boolean remission to 0.79 for DAS28-ESR remission and DAS28-CRP remission.

**Table 2:** Ability of the MBDA score to differentiate between clinical remission and non-remission for different definitions of remission

Baseline MBDA score vs baseline remission criteria	AUROC	95% CI
DAS28-ESR (*N* = 140)	0.75	0.67–0.83
DAS28-CRP (*N* = 136)	0.69	0.60–0.78
SDAI (*N* = 135)	0.70	0.61–0.79
CDAI (*N* = 144)	0.68	0.59–0.77
ACR/EULAR Boolean (*N* = 136)	0.70	0.60–0.79
Baseline MBDA score vs IR/SR criteria	AUROC	95% CI
DAS28-ESR (*N* = 144)	0.73	0.61–0.85
DAS28-CRP (*N* = 144)	0.74	0.64–0.83
SDAI (*N* = 144)	0.71	0.62–0.80
CDAI (*N* = 145)	0.69	0.60–0.78
ACR/EULAR Boolean (*N* = 144)	0.65	0.56–0.74
tiMBDA score vs IR/SR criteria	AUROC	95% CI
DAS28-ESR (*N* = 147)	0.79	0.70–0.91
DAS28-CRP (*N* = 147)	0.79	0.70–0.88
SDAI (*N* = 147)	0.73	0.64–0.82
CDAI (*N* = 148)	0.71	0.62–0.80
ACR/EULAR Boolean (*N* = 147)	0.65	0.56–0.75

Area under the receiver operating curves (AUROCs) for the discriminative ability of the MBDA score. The upper section shows the ability of baseline MBDA score to differentiate between baseline clinical remission and no remission at baseline. The middle and lower sections show the ability of baseline MBDA score and the time-integrated MBDA (tiMBDA) score over 6 months, respectively, to differentiate between clinical intermittent or sustained remission (IR/SR) and sustained non-remission over 1 year

### Clinical remission and biomarker levels at baseline

At baseline, 96 (65%) patients were in DAS28-ESR remission, 75 (51%) in DAS28-CRP remission, 59 (40%) in SDAI remission, 64 (43%) in CDAI remission and 37 (25%) fulfilled the ACR/EULAR Boolean remission criteria; 40 (27%) patients were in the LDAS group, meaning they did not fulfil any of the remission criteria. Table 3 shows that the baseline MBDA score was significantly lower in each remission group when compared to the LDAS group, with a trend across remission groups for the MBDA score to be lower for those with more stringent remission criteria (i.e. smaller *n* values). Among the 12 MBDA component biomarkers at baseline, IL-6, leptin, SAA and CRP were each significantly lower in all five baseline remission groups when compared to the LDAS group; resistin, YKL-40, TNFR1 and VCAM1 were significantly lower in DAS28-ESR baseline remission group; resistin, YKL-40 and VCAM1 were significantly lower in the DAS28-CRP baseline remission group and VCAM1 was significantly lower in CDAI and SDAI baseline remission groups (Table 3).

**Table 3:** Baseline biomarker concentrations and MBDA scores for patients in LDAS and remission at baseline

Baseline Biomarkers	LDAS (***n*** = 40)	DAS28-ESR remission (***n*** = 96)	DAS28-CRP remission (***n*** = 74)	CDAI remission (***n*** = 63)	SDAI remission (***n*** = 59)	ACR/EULAR Boolean remission (***n*** = 37)
EGF (pg/ml)	245 (160, 331)	254 (177, 368)	249 (160, 366)	248 (158, 359)	243 (157, 362)	249 (180, 373)
IL-6 (pg/ml)	13.65 (9.89, 24.35)	7.14 (5.56, 13.21)****	8.09 (5.67, 12.62)****	7.89 (5.58, 13.21)****	6.97 (5.41, 11.79)****	6.29 (4.41, 9.33)****
Leptin (ng/ml)	25 (6.3, 38)	8 (4, 17)*	8 (4, 16) *	7.00 (3.54, 14.10)*	6.33 (3.50,13.87)***	6.33 (3.50, 11.74)**
MMP-1 (ng/ml)	6.62 (4.41, 10.48)	6.80 (4.93, 9.49)	7.30 (5.07, 10.63)	7.52 (5.13, 10.78)	7.57 (5.19, 10.93)	7.47 (5.25, 1.09)
MMP-3 (ng/ml)	26 (17, 40)	26 (20, 38)	26 (17, 35)	26 (17, 37)	27 (17, 35)	27 (17, 40)
Resistin (ng/ml)	8.72 (6.40, 12.01)	7.04 (5.78, 8.72)**	7.11 (5.84, 9.54)*	7.37 (6.20, 9.69)	7.32 (6.04, 9.85)	7.92 (6.04, 9.54)
SAA (μl/ml)	2.82 (1.63, 6.17)	1.32 (0.69, 2.49)***	1.22 (0.65, 2.13)****	1.52 (0.65, 2.13)***	1.20 (6.43, 2.06)****	1.20 (0.65, 2.03)****
TNFR1 (ng/ml)	1.76 (1.37, 2.16)	1.50 (1.32, 1.92)*	1.51 (1.35, 1.99)	1.52 (1.33, 2.01)	1.51 (1.31, 1.99)	1.64 (1.34, 1.99)
VCAM1 (ng/ml)	587 (469, 732)	518 (433, 587)**	519 (440, 588)*	520 (425, 609)*	521 (430, 621)*	531 (457, 621)
VEGF (pg/ml)	294 (211, 365)	240 (166, 374)	246 (173, 408)	258 (174, 407)	247 (173, 407)	275 (176, 407)
YKL40 (ng/ml)	65 (42, 97)	51 (34, 70)*	56 (36, 92)*	53 (35, 102)	52 (34, 98)	56 (35, 92)
CRP (mg/ml)	6.41 (3.23, 14.10)	1.49 (0.61, 2.83)****	1.57 (0.61, 2.70)****	1.40 (0.78, 2.63)****	1.40 (0.56, 2.49)****	1.40 (0.44, 2.49)****
MBDA score (1–100)	39 (31, 50)	26 (16, 37)****	26 (16, 35)****	24 (16, 35)****	22 (16, 33)****	20 (15, 32)****

Comparison of the low disease activity state (LDAS, defined as not fulfilling any clinical remission criteria) group to the DAS28-ESR, DAS28CRP, CDAI, SDAI and Boolean remission groups at baseline. LDAS was defined as patients who were not in remission by any definition at baseline

Values are median (IQR). Levels of significance determined by Mann Whitney test. *p < 0.05, **p < 0.01, ***p < 0.001, ****p < 0.0001

### Baseline biomarker levels and clinical remission over 1 year

Over 1 year, 14 (9%) patients did not fulfil any remission criteria at any time point (PDA group). Sixty-two (42%) and 69 (47%) patients achieved intermittent (IR) and sustained (SR) DAS28-ESR remission, respectively. Sixty-eight (46%) and 43 (29%) of patients achieved DAS28-CRP IR and DAS28-CRP SR, respectively; 67 (45%) and 29 (20%) of patients achieved SDAI IR and SDAI SR, respectively; 65 (44%) and 31 (21%) of patients achieved CDAI IR and CDAI SR, respectively; and 52 (35%) and 14 (9%) achieved ACR/EULAR Boolean IR and SR, respectively. A statistically significant trend of decreasing baseline MBDA score was observed across the PDA/IR/SR groups defined by DAS28-ESR, SDAI or ACR/EULAR Boolean remission (Table 4). There was also a statistically significant trend of decrease in baseline IL-6, leptin, SAA and CRP concentrations across the PDA/IR/SR groups using these 3 remission criteria (Table 4). Similar results were seen for DAS28-CRP remission (except that SAA was not statistically significant and VEGF was) and for CDAI remission (Supplementary Table 1).

**Table 4:** Baseline biomarker concentrations and MBDA scores in the groups with persistent disease activity, intermittent remission and sustained remission over 12 months

	PDA (***n*** = 14)	DAS28-IR (***n*** = 62)	DAS28-SR (***n*** = 69)	***p*** value	SDAI-IR (***n*** = 67)	SDAI-SR (***n*** = 29)	***p*** value	ACR/EULAR Boolean-IR (***n*** = 52)	ACR/EULAR Boolean-SR (***n*** = 14)	***p*** value
**EGF (pg/ml)**	287 (162, 368)	235 (181, 334)	246 (148, 368)	ns	245 (148, 356)	249 (145, 339)	ns	228 (154, 338)	253 (145, 397)	ns
**IL-6 (pg/ml)**	14 (10, 24)	12 (7, 21)	8 (5, 12)	0.0001	8.46 (5.97, 13.21)	6.16 (4.24, 10.21)	0.0008	7.13 (5.41, 12.00)	7.79 (5.58, 9.33)	0.0056
**Leptin (ng/ml)**	25 (10, 35)	16 (6, 33)	7 (4, 16)	0.0006	11 (4, 24)	6 (4, 11)	0.009	7.87 (4.12, 2.34)	6.74 (3.44, 14)	0.031
**MMP-1 (ng/ml)**	5.10 (3.82, 8.99)	6.79 (4.87, 9.34)	7.08 (3.54, 9.61)	ns	7.26 (5.05, 10.49)	6.84 (4.53, 9.12)	ns	6.62 (5.06, 10.01)	7.95 (4.60, 8.71)	ns
**MMP-3 (ng/ml)**	27 (14, 43)	24 (17, 34)	27 (19, 37)	ns	27 (21, 40)	20 (15, 32)	ns	27 (16, 34)	19 (15, 33)	ns
**Resistin (ng/ml)**	9.10 (6.47, 12.49)	7 (5, 9)	8 (6,9)	ns	7.01 (5.91, 8.44)	7.99 (5.84, 9.88)	ns	7.02 (5.87, 8.94)	8.14 (7.58, 10.25)	ns
**SAA (ug/ml)**	2.82 (1.84, 8.75)	1.73 (1.06, 3.74)	1.23 (0.64, 2.51)	0.0012	1.69 (0.70, 3.10)	1.09 (0.64, 1.70)	0.0013	1.54 (0.85, 2.47)	1.14 (6.43, 2.21)	0.0062
**TNFR1 (ng/ml)**	1.66 (1.39, 2.11)	1.56 (1.35, 1.94)	1.50 (1.32, 1.99)	ns	1.51 (1.32, 1.94)	1.50 (1.31, 1.90)	ns	1.55 (1.35, 2.01)	1.73 (1,31, 2.06)	ns
**VCAM1 (ng/ml)**	587 (528, 693)	524 (409, 652)	533 (457, 621)	ns	509 (421, 621)	521 (457, 596)	ns	532 (427, 626)	511 (458, 596)	ns
**VEGF (pg/ml)**	330 (260, 478)	263 (190, 368)	247 (173, 393)	ns	247 (186, 372)	215 (150, 408)	ns	218 (154, 385)	208 (170, 330)	ns
**YKL40 (ng/ml)**	70 (45, 98)	57 (38, 86)	53 (36, 92)	ns	50 (36, 69)	48 (32, 127)	ns	55 (33, 92)	60 (48, 13)	ns
**CRP (mg/ml)**	6.63 (4.99, 10.50)	2.98 (1.25, 7.39)	1.40 (0.61, 2.70)	< 0.0001	1.65 (1.03, 3.50)	1.32 (0.35, 2.34)	< 0.0001	2.06 (0.79, 2.98)	1.15 (0.21, 2.49)	< 0.0001
**MBDA score**	40 (31, 50)	34 (21, 43)	23 (16, 35)	< 0.0001	29 (18, 35)	17 (15, 35)	0.0002	26 (17, 37)	21.5 (15, 37)	0.0025

Comparison of baseline values in the persistent disease activity (PDA, i.e., no remission by any criteria at any visit) group, the intermittent remission (IR) group and the sustained remission (SR) group, based on DAS28-ESR, SDAI and ACR/EULAR Boolean definitions of remission

Values are median (IQR). Values across the PDA/IR/SR groups were assessed using Jonckheere-Terpstra trend test

### Time-integrated biomarker levels and clinical remission over 1 year

Median (IQR) time-integrated (ti) values over 6 months were determined for the MBDA score and its biomarkers in the PDA, IR and SR groups (Table 5). Statistically significant trends of decreasing tiMBDA score and decreasing tiIL-6, tileptin, tiSAA and tiCRP values were observed across the PDA/IR/SR groups for each of the remission criteria. There was also a significant trend of decreasing tiVGEF values across the PDA/IR/SR groups for the DAS28-ESR and ACR/EULAR Boolean definitions of remission. The *p* values for the test of trend across the PDA, IR and SR groups were smaller for the tiMBDA score (Table 5) than the baseline MBDA score (Table 4), using the SDAI and ACR/EULAR Boolean definitions of remission. Smaller *p* values were also noted for tileptin and tiSAA, compared to the respective baseline values using all 3 remission criteria. Similar trends of tileptin, tiSAA, tiCRP, tiIL-6 and tiMBDA scores were observed using the DAS28-CRP and CDAI remission criteria (Supplementary Table 2).

**Table 5:** Time-integrated biomarker concentrations and MBDA scores in the groups with persistent disease activity, intermittent remission and sustained remission over 12 months

	PDA (***n*** = 14)	DAS28-IR (***n*** = 62)	DAS28-SR (***n*** = 69)	***p*** value	SDAI-IR (***n*** = 67)	SDAI-SR (***n*** = 29)	***p*** value	ACR/EULAR Boolean-IR (***n*** = 52)	ACR/EULAR Boolean-SR (***n*** = 14)	***p*** value
**tiEGF (pg/ml)**	208 (202, 368)	276 (175, 358)	257 (149, 359)	ns	275 (161, 358)	286 (146, 329)	ns	243 (161, 324)	275.71 (117.88, 328.72)	ns
**tiIL-6 (pg/ml)**	15.82 (13.07, 38.96)	12.66 (7.46, 17.85)	8.06 (5.88, 11.09)	< 0.0001	8.77 (6.72, 14)	7.89 (5.13, 11.06)	0.0009	8.37 (6.19, 11.96)	8.23 (5.88, 10.18)	0.0013
**tiLeptin (ng/ml)**	18.89 (97.78, 33.32)	16.99 (6.28, 35.78)	7.12 (3.82, 14.39)	0.0005	11.82 (4.91, 26.01)	6.38 (3.82, 9.99)	0.0038	8.04 (4.75, 23.76)	6.80 (3.32, 10.24)	0.015
**tiMMP-1(ng/ml)**	6.13(4.26, 8.86)	7.23 (4.92, 9.30)	6.87 (4.45, 9.93)	ns	7.23 (4.92, 9.89)	6.51 (4.52, 9.93)	ns	6.69 (4.95, 9.61)	7.67 (5.00, 9.41)	ns
**tiMMP-3 (ng/ml)**	28.99 (13.81, 39.76)	23.02 (17.38, 33.75)	27.29 (20.97, 36.47)	ns	27.55 (19.50, 38.74)	22.22 (15.46, 33.18)	ns	25.99 (17.42, 33.96)	22.36 (15.46, 34.76)	ns
**tiResistin (ng/ml)**	11.66 (80.60, 13.07)	7.57 (6.15, 9.50)	7.56 (6.61, 9.17)	ns	7.52 (6.29, 8.75)	8.42 (6.56, 9.88)	ns	7.47 (6.41, 9.20)	8.61 (6.63, 10.15)	ns
**tiSAA (ug/ml)**	3.99 (3.20, 8.47)	2.01 (1.00, 4.43)	1.29 (0.99, 4.44)	0.0001	1.84 (0.93, 3.10)	1.06 (0.74, 2.16)	0.0002	1.71 (0.90, 2.59)	0.94 (0.75, 2.28)	0.0004
**tiTNFR1 (ng/ml)**	1.79 (1.47, 2.10)	1.56 (1.37, 2.02)	1.56 (1.37, 1.97)	ns	1.53 (1.35, 1.97)	1.51 (1.36, 1.82)	ns	1.58 (1.37, 1.98)	1.70 (1.28, 2.00)	ns
**tiVCAM1 (ng/ml)**	590 (476, 652)	527 (427, 618)	490 (437, 571)	ns	493 (433, 578)	486 (429, 613)	ns	495 (441, 594)	479 (397, 636)	ns
**tiVEGF (pg/ml)**	338 (267, 487)	262 (198, 370)	264 (182, 409)	0.042	240 (182, 381)	226 (154, 423)	ns	212 (163, 378)	213 (154, 297)	0.042
**tiYKL40 (ng/ml)**	79.23 (60.80, 143.49)	60.56 (40.67, 102.18)	54.90 (34.77, 99.67)	ns	54.96 (35,25, 82.49)	48.78 (34.62, 120.90)	ns	53.50 (33.45, 100)	59.06 (41.76, 118.82)	ns
**tiCRP (mg/ml)**	8.38 (4.25, 12.70)	3.52 (1.64, 7.75)	1.73 (6.53, 3.47)	< 0.0001	1.98 (1.05, 1.35)	0.98 (0.65, 3.31)	0.0001	2.09 (0.9, 3.75)	0.93 (0.52, 2.70)	< 0.0001
**tiMBDA score (0–100)**	43 (36, 51)	33 (22, 41)	24 (17, 33)	< 0.0001	28 (20, 34)	22 (16, 33)	< 0.0001	25 (20, 34)	22 (15, 33)	< 0.0001

Comparison of time-integrated (ti) values in the persistent disease activity (PDA, i.e. no remission by any criteria at any visit) group, the intermittent remission (IR) and the sustained remission (SR) group, based on DAS28-ESR, SDAI and ACR/EULAR Boolean definitions of remission. Values are medians (IQR). Values across the PDA/IR/SR groups were assessed using Jonckheere-Terpstra trend test

**Fig. 2:**
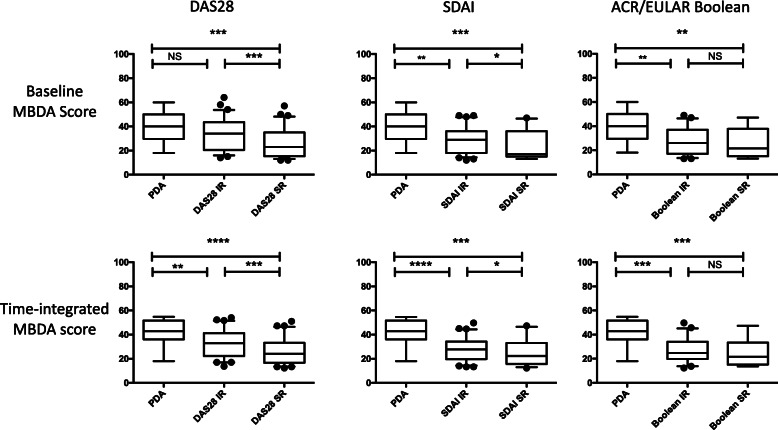
MBDA Scores in PDA, IR and SR states. **a–c** Baseline MBDA scores. **d–f** Time-integrated MBDA scores. Patients were grouped with remission defined in **a** and **d** by DAS28, in **b** and **e** by SDAI and in **c** and **f** by ACR/EULAR Boolean criteria. Patient numbers in each group are the same as in Tables 4 and 5. Level of significance determined by Mann-Whitney *U* test. Values expressed as medians with IQR. PDA, persistent disease activity, i.e. no remission by any criteria at any visit; IR, intermittent remission; SR sustained remission. Levels of significance determined by Mann-Whitney test (NR vs IR, IR vs SR, NR vs SR). ns = *p* > 0.05, *p < 0.05, **p < 0.01, ***p < 0.001, ****p < 0.0001

To examine the results in Tables 4 and 5 in greater detail, pairwise comparisons were made between baseline MBDA scores (Fig. 2 top) or tiMBDA scores (Fig. 2 bottom) among the PDA, IR and SR groups, as defined by DAS28-ESR, CDAI and ACR/EULAR Boolean remission criteria. All of these 18 comparisons were statistically significant except for three: baseline MBDA score in the PDA versus DAS28-ESR IR groups, and baseline MBDA score and tiMBDA score in the ACR/EULAR Boolean IR vs. SR groups.

## Discussion

Our study found that, in a cohort of patients with DAS28-ESR < 3.2 at baseline, i.e., patients in low clinical disease activity including remission, the MBDA score was able to differentiate between remission and non-remission, as defined by several clinical composite criteria. This ability was observed in cross-sectional and predictive analyses across five definitions of remission, and it existed despite the considerable molecular heterogeneity seen in this patient cohort. Similarly, we identified individual MBDA biomarkers (IL-6, leptin, SAA, CRP) which differentiated, cross-sectionally, between remission at baseline and the absence of remission by any definition (LDAS). Furthermore, we found that MBDA score, IL-6, leptin, SAA and CRP at baseline were each able to predict intermittent or sustained remission (IR/SR) over 12 months. The average of 3 measurements obtained over 6 months, i.e., the time-integrated assessment, performed slightly better as a predictor than a single baseline measurement.

The clinical validity of the MBDA score was originally established by demonstrating significant association between the MBDA score and DAS28-CRP in RA patients that were heterogeneous in terms of autoantibody status, disease activity and RA treatment [18]. Cross-sectional analyses of a cohort with a wide range of clinical disease activity showed that the MBDA score correlated with DAS28-CRP and discriminated between patients in the low versus moderate-to-high DAS28-CRP categories, with AUROCs of 0.77 and 0.70 for seropositive and seronegative patients, respectively [18]. Change in MBDA score discriminated between responders and non-responders by DAS28-CRP (AUROC = 0.77, *p* = 0.002) and by ACR50 (AUROC = 0.69, *p* = 0.03) [18]. A recent systematic review found that the MBDA score demonstrated moderate convergent validity with DAS28-CRP and DAS28-ESR and weaker correlations with SDAI, CDAI and RAPID3 [24]. Our study included only patients with DAS28-ESR < 3.2 at baseline. Thus, the cross-sectional finding that the baseline MBDA score discriminated between remission and non-remission, using DAS28-ESR, DAS28-CRP, SDAI, CDAI and ACR/EULAR Boolean criteria (Table 2, AUROCs 0.69–0.75), is consistent with results of the MBDA score validation studies. It also complements them by showing that the MBDA score performed well within the lower end of the clinical disease activity spectrum. Our predictive finding, that the baseline MBDA score and the tiMBDA score discriminated between remission (IR/SR) and non-remission (PDAS) at 1 year, is novel.

The DRESS study enrolled RA patients in low disease activity or remission, with the primary objective of evaluating flare following TNFi tapering. In the usual care arm of DRESS, where patients maintained their TNFi treatment, it was found that the baseline MBDA score was significantly associated with major flare over the next 12 months [25]. This result is in keeping with our finding that the MBDA score at baseline was predictive of remission over 1 year and was lowest in patients who achieved sustained remission by the more stringent SDAI or ACR/EULAR Boolean definitions.

In a previous report, we did not find that the baseline MBDA score was predictive of flare overall [23]. However, a sensitivity analysis of patients who flared clinically with an increase in MBDA score to > 44 (high disease activity) demonstrated a relationship between baseline MBDA score and risk of flare, suggesting that the MBDA score was more predictive of objective flares than flares dominated by subjective changes [23]. In RETRO, a study of RA patients in stable remission who underwent tapering of all DMARDs simultaneously, baseline MBDA scores in the moderate or high category were significantly associated with a higher relapse rate [26]. In POET, a study of RA patients in stable low disease activity/remission who discontinued anti-TNF treatment, a high baseline MBDA score was independently predictive of disease relapse within 12 months [27]. By contrast, MBDA score at baseline was not associated with flare following treatment reduction in the DRESS [25] or STRASS studies [28]. Flares that followed treatment reduction in these studies should be compared cautiously with our study, in which treatment was maintained. Our present study is distinct from these others in that we reported sustained remission and not flare over time. In addition, we also averaged three measurements over 6 months to predict remission outcomes. Our finding that these time-integrated values for the MBDA score and for the individual biomarkers tended to be more statistically significant than baseline values as predictors of sustained remission over 1 year is novel and requires confirmation in other studies.

This study found that patients in low clinical disease activity/remission (DAS28-ESR < 3.2) were heterogenous at the molecular level. It implies that more than one biomarker is likely to be required to accurately identify patients in remission. Intermittent and sustained remission were associated with lower baseline MBDA scores and lower concentrations of IL-6, leptin, SAA and CRP, compared with patients who never achieved remission. This finding suggests that biochemical evidence of inflammation exists in patients with low levels of clinical disease activity.

A strength of our study is that it acquired data from clinical assessments every 3 months over a 12-month period in a relatively large population of RA patients with similar clinical phenotypes. As this study has demonstrated, patients in clinically stable low disease activity can be heterogeneous at a molecular level, and their disease activity can change over time. Therefore, a stringent definition of sustained remission was used in this study, requiring remission every 3 months over 1 year. The original ACR remission criteria described by Pinals et al. in 1981 [29] concluded that ‘complete’ RA remission indicates the ‘total absence of articular and extraarticular inflammation and immunological activities’. Despite advances in therapies, this goal is still difficult to achieve. The criteria also incorporated time into the definition of remission, namely, remission over 2 consecutive months. Other definitions of sustained remission have used longer periods of time. Jayakumar et al. [30] evaluated sustained remission over 5 years and Prince et al. 7 years [31]. Currently, there is no consensus on what the optimal length of time should be. For the REMIRA study, 1 year was chosen to define sustained remission in a way that adequately assesses the stability of disease activity and is feasible in clinical practice.

Limitations of the study include the relatively small number of patients in sustained remission, particularly in the group meeting the ACR/EULAR Boolean definition and in the group with no remission at any time point, i.e. the PDA group. Secondly, because the different remission groups contained overlapping populations, it was not possible to formally compare them to each other. Thirdly, ACPA status was not analysed as a predictor of remission in REMIRA because the focus of this study was the MBDA score and its biomarkers and because ACPA data were incomplete. Among the studies of treatment reduction that evaluated the MBDA score, ACPA was associated with flare in RETRO [26] but not DRESS [32], POET [33] or STRASS [34]. Lastly, BMI data was not collected in this study and the MBDA scores were not adjusted for adiposity. Thus, obesity may have affected univariate analyses of leptin and the MBDA score [35], although it seems unlikely that obesity alone could explain the large differences observed between patients who were in remission vs. those who were not.

## Conclusion

This study found that, in a cohort of RA patients who were in low clinical disease activity states, including remission, serum protein biomarkers may be useful for defining remission states and predicting sustained remission. Even within the small spectrum of disease activity that we studied, the MBDA score and some of its analytes differentiated between similar levels of clinical disease activity at baseline and, with a single measurement at baseline or multiple measurements from baseline to 6 months, predicted remission outcomes at 1 year. These findings highlight the importance of frequent longitudinal follow-up of patients who have achieved low disease activity or clinical remission and the potential value of repeated measurements to evaluate stability of clinical disease activity over time.

## Supplementary information

**Additional file 1: Supplementary Table 1.** Baseline biomarker concentrations and MBDA scores in the groups with persistent disease activity, intermittent remission and sustained remission over 12 months*. Comparison of baseline values in the persistent disease activity (PDA,* i.e.*, no remission by any criteria at any visit) group, the intermittent remission (IR) group and the sustained remission (SR) group, based on DAS28-CRP and CDAI remission groups. Values are median (IQR). Values across the PDA/IR/SR groups were assessed using Jonckheere-Terpstra trend test.*

**Additional file 2: Supplementary Table 2.** Time-integrated biomarker concentrations and MBDA scores in the groups with persistent disease activity, intermittent remission and sustained remission over 12 months*.* Comparison of time-integrated (ti) values in the persistent disease activity (PDA, i.e., no remission by any criteria at any visit) group, the intermittent remission (IR) and the sustained remission (SR) group, *based on DAS28-CRP and CDAI remission groups. Values are median (IQR). Values across the PDA/IR/SR groups were assessed using Jonckheere-Terpstra trend test.*

## Data Availability

The datasets used and/or analysed during the current study are available from the corresponding author on reasonable request.

## Abbreviations

ACR: American College of Rheumatology
ACPA: Anti-citrullinated protein antibodies
anti-TNF: Anti-tumour necrosis factor
AUROC: Area under the receiver operating characteristic curve
CDAI: Clinical Disease Activity Index
CRP: C-reactive protein
csDMARDs: Conventional synthetic disease-modifying antirheumatic drugs
DAS28: Disease activity score-28 joints
DRESS: Dose reduction strategy of subcutaneous TNF inhibitors in rheumatoid arthritis study
EGF: Epidermal growth factor
ESR: Erythrocyte sedimentation rate
EULAR: European league against Rheumatism
IL6: Interleukin-6
IQR: Interquartile range
IR: Intermittent remission
LDAS: Low disease activity
LOCF: Last-observation-carried-forward
MBDA: Multi-biomarker disease activity
MMP1: Matrix metalloproteinases 1
MMP3: Matrix metalloproteinases 3
OPERA: Optimized treatment algorithm for patients with early rheumatoid arthritis study
PDA: Persistent disease activity
RA: Rheumatoid arthritis
REMIRA: Remission in RA Study
RF: Rheumatoid factor
SAA: Serum amyloid-A
SDAI: Simple disease activity index
SJC28: Swollen joint count 28
SR: Sustained remission
ti: Time-integrated
tiMBDA score: Time-integrated multi-biomarker disease activity score
TJC28: Tender joint count 28
TNFR1: Tumour necrosis factor receptor 1
VAS: Visual analogue score
VCAM1: Vascular cell adhesion molecule 1
VEGF: Vascular endothelial growth factor
