# Supplementary material for: A multi-biomarker disease activity score can predict sustained remission in rheumatoid arthritis
Source: Arthritis Res Ther. 2020 Jun 24;22:158. doi: 10.1186/s13075-020-02240-w (PMC7313155; doi:10.1186/s13075-020-02240-w)
Supplement: Supplementary file 1 — Additional file 1: Supplementary Table 1. Baseline biomarker concentrations and MBDA scores in the groups with persistent disease activity, intermittent remission and sustained remission over 12 months. Comparison of baseline values in the persistent disease activity (PDA, i.e., no remission by any criteria at any visit) group, the intermittent remission (IR) group and the sustained remission (SR) group, based on DAS28-CRP and CDAI remission groups. Values are median (IQR). Values across the PDA/IR/SR groups were assessed using Jonckheere-Terpstra trend test. [file 13075_2020_2240_MOESM1_ESM.docx]

Supplementary Table 1: Baseline biomarker concentrations and MBDA scores in the groups with persistent disease activity, intermittent remission and sustained remission over 12 months

*Comparison of baseline values in the persistent disease activity (PDA, i.e., no remission by any criteria at any visit) group, the intermittent remission (IR) group and the sustained remission (SR) group, based on DAS28-CRP and CDAI remission groups.*

*Values are median (IQR). Values across the PDA/IR/SR groups were assessed using Jonckheere-Terpstra trend test.*

|  | **PDA (n=14)** | **DAS28CRP-IR (n=68)** | **DAS28CRP-SR (n=43)** | **p**  **value** | **CDAI-IR (n=65)** | **CDAI-SR (n=31)** | **p**  **value** |
| --- | --- | --- | --- | --- | --- | --- | --- |
| **EGF (pg/ml)** | 287 (162, 368) | 271 (182, 371) | 244 (145, 333) | ns | 245 (148, 342) | 265 (145, 362) | ns |
| **IL-6 (pg/ml)** | 14 (10, 24) | 7 (4, 13) | 8 (5, 12) | 0.0012 | 8.80 (5.97, 13.42) | 6.19 (4.24, 10.21) | 0.0006 |
| **Leptin (ng/ml)** | 25 (10, 35) | 14 (4, 25) | 8 (4, 15) | 0.02 | 11 (4, 23) | 6 (4, 12) | 0.01 |
| **MMP-1 (ng/ml)** | 5.10 (3.82, 8.99) | 7 (4.61, 10.78) | 6.81 (4.59, 9.12) | ns | 7.30 (5.07, 10.49) | 6.84 (4.49, 9.12) | ns |
| **MMP-3 (ng/ml)** | 27 (14, 43) | 26 (21, 37) | 26 (16, 33) | ns | 27 (21, 40) | 20 (15, 32) | ns |
| **Resistin (ng/ml)** | 9.10 (6.47, 12.49) | 7 (6, 9) | 8 (6,10) | ns | 7.11 (6.04, 8.44) | 7.99 (5.84, 9.88) | ns |
| **SAA (ug/ml)** | 2.82 (1.84, 8.75) | 1.52 (0.76, 2.53) | 1.28 (0.90, 2.44) | ns (0.06) | 1.72 (0.81, 3.15) | 1.14 (0.58, 2.03) | 0.0015 |
| **TNFR1 (ng/ml)** | 1.66 (1.39, 2.11) | 1.54 (1.33, 1.98) | 1.50 (1.31, 1.92) | ns | 1.52 (1.33, 1.99) | 1.50 (1.31, 1.90) | ns |
| **VCAM1 (ng/ml)** | 587 (528, 693) | 513 (425, 603) | 521 (444, 627) | ns | 530 (430, 624) | 519 (449, 596) | ns |
| **VEGF (pg/ml)** | 330 (260, 478) | 263 (196, 380) | 226 (150, 393) | 0.048 | 246 (186, 370) | 216 (148, 408) | ns |
| **YKL40 (ng/ml)** | 70 (45, 98) | 56 (36, 74) | 48 (35, 121) | ns | 52 (38, 70) | 48 (32, 127) | ns |
| **CRP (mg/ml)** | 6.63 (4.99, 10.50) | 1.73 (1.03, 4.26) | 1.63 (0.44, 2.70) | 0.0007 | 1.65 (1.03, 3.50) | 1.32 (0.35, 2.34) | 0.0001 |
| **MBDA score** | 40 (31, 50) | 29 (18, 37) | 26 (15, 35) | 0.004 | 31 (18, 37) | 18 (15, 35) | 0.0001 |
